# Finding New Collaboration Models for Enabling Neglected Tropical Disease Drug Discovery

**DOI:** 10.1371/journal.pntd.0002866

**Published:** 2014-07-03

**Authors:** Michael P. Pollastri

**Affiliations:** Department of Chemistry & Chemical Biology, Northeastern University, Egan Research Center, Boston, Massachusetts, United States of America; Swiss Tropical and Public Health Institute, Switzerland

Neglected tropical diseases (NTDs) have seen a welcome bolstering of activities focused on discovery of new therapies for these diseases. By and large, NTD drug discovery happens in the nonprofit sector—in academic laboratories and in public–private partnerships—though there has also been a significant and tangible influx of data and research contributions from the for-profit biopharmaceutical industry. Sets of screening data against the parasites that cause Chagas disease and African sleeping sickness have been released to the public via ChemBL (https://www.ebi.ac.uk/chemblntd), Collaborative Drug Discovery (http://www.collaborativedrug.com), and PubChem (https://pubchem.ncbi.nlm.nih.gov), and a fair quantity of these data have been produced by the pharmaceutical industry, many times in collaboration with groups in the nonprofit or academic environment. These initial public releases have begun to enable credible drug discovery for tropical diseases, particularly when taken together with new collaborative opportunities with industry that provide access to state-of-the-art drug discovery and development capabilities. These facilities include the Tres Cantos Open Lab initiative [1], therapeutics development resources at the National Institute of Allergy and Infectious Diseases [2], and compound screening sets now made available for testing against other pathogens, such as the Malaria Box [3]. Thus, perhaps there has never been a better time to be performing hit-to-lead and lead optimization drug discovery for NTDs.

Some of the best practices in industrial drug discovery, which include careful compound design, streamlined synthesis, compound assessment via a well-defined testing cascade, plus informatics implementation to interpret the experimental results, are now being applied to NTD drug discovery. This environment has produced credible, early-stage drug discovery programs that are more likely to produce new therapies for NTDs in the coming years and fill the pipelines within product development partnerships.

The for-profit industrial drug discovery engine is tuned for working on indications that can both recoup research costs and draw profits from drug sales, and, as a result, careful protection of trade secrets and heavy use of patenting predominates, though there are increasing efforts to pull back the veil of secrecy on precompetitive aspects of drug discovery (such as predictive models or screening technologies) [4]. One needs to be cautious to prevent practices of secrecy from pervading these new “industrialized” NTD drug discovery efforts. Excitingly, many working in this area are industrially experienced, which allows them to bring a different mindset to academic drug discovery. One knock-on effect of this, however, is that many of these individuals (myself included!) often adopt the “closed” drug discovery process, simply by habit or an overabundance of caution, without careful consideration about why this information is being protected in the first place for indications where little, if any, profit can be made.

There are also additional (real or perceived) disincentives for wider data sharing in the academic environment. First, research results in this environment are mostly reported via journal publication, arguably the central currency of academic productivity and hence important for obtaining funding and visibility. Publishing typically requires the construction of a complete story of a hypothesis-driven project. In drug discovery, a complete story often can require many years of research, and always includes negative results (often deemed “unpublishable”). Such results include, for example, inactive or toxic compounds, compounds with poor metabolic profiles, etc. Such compounds are often not further pursued, yet such data remains pivotal for driving a drug discovery project. Molecular modeling and computational chemistry efforts strongly benefit from such “negative” data, as well. In the industrial world, many companies actively discourage publication of terminated drug discovery projects to reduce the likelihood of providing a competitor any kind of advantage that such publication could provide.

Once the story is deemed complete and impactful enough to publish, several additional months may pass before publication. In short, the time between experimental result and data sharing is too long for others in the field to use these results for their own projects in real time, and the general lack of negative data can reduce the impact of these publications.

Another potential disincentive for wider data sharing is the ever-increasing difficulty in securing competitive research funding, for which strong preliminary data is pivotal. There are fears (not completely unfounded!) that sharing one's preliminary results with others in the field could potentially inform competing labs' own grant applications by direct or indirect use of this information.

This situation brings several questions to mind: Since there are more laboratories working on drug discovery for NTDs, how much effort is being wastefully duplicated during these months and years between discovery and communication? (Note the difference between “duplication,” which is wasteful, and “replication” which is important to ensure scientific robustness). Such duplication is not unique to NTDs, but so few resources are invested in NTD drug discovery [5] that it should be avoided as a priority. How many opportunities for collaboration and load sharing have been lost? Could there be “negative” data that could be critically important to someone else's drug discovery program or computational models, yet that will never see the light of day? Indeed, such data needs to be collated and curated for effective mining efforts, which is often deemed to be an ineffective use of time. Perhaps most importantly: *What is actually gained by secrecy of experimental results during drug discovery for NTDs?*

There have been some new collaboration models established in recent years that involve “open science,” and a recent descriptive case study evaluating and comparing two particular drug discovery projects in tuberculosis (TB) and schistosomiasis will provide the reader some helpful context as to why this is something to which the NTD field should aspire [6]. In the malaria drug discovery field, Dr. Matthew Todd has launched a sizable “open source” drug discovery campaign (Open Source Drug Discovery-Malaria [OSDD-Malaria]) that is focused on coordinated follow-up of the GlaxoSmithKline (GSK) high-throughput screening (HTS) hits described in 2010 [7]. In this program, experimental observations, data, and ideas are shared openly; compounds are synthesized by anyone worldwide who wishes to contribute to the effort; and screening data is generated and shared in real time via the internet. Anyone who wishes to view this information and/or contribute to the ongoing project by generating ideas and performing experiments is welcome. By all accounts, the model appears to be proceeding very well—one can easily discern the overall project status from the project wiki page [8] and join the program. This is but one example of open science that NTD drug hunters may look towards, and, through these examples, there is increasing sense that these are indications that likely do not require an air-tight intellectual property position. To wit: Medicines for Malaria Venture, arguably the premier and most influential malaria drug development organization, is an active participant in the OSDD-Malaria program, which lends credence to the value of open science for such work.

That success aside, not all investigators in NTD drug discovery are prepared just yet to openly share all their data and ideas in real time with the general public, sometimes out of habit, sometimes driven by the desire to file patents for new drugs for NTDs, or sometimes out of a fear of being scooped. Perhaps this feeling is most acutely felt by former pharmaceutical industry scientists who are transitioning into the nonprofit environment and wish to work on NTD drug discovery, a difficulty to which I can personally attest. Furthermore, individual organizations may have data sharing restrictions placed upon them by their funders (who may, in turn, be expecting some modicum of financial return upon commercialization of a new product resulting from their funding). There is, therefore, a need for a mechanism by which data and ideas can be shared with some measure of confidentiality. In addition, in contrast to the OSDD-Malaria program described above, which is a coordinated drug discovery effort focused on specific chemotypes identified in the GSK HTS campaign, not all research groups wish to collaborate in this kind of coordinated environment. Nonetheless, these uncoordinated programs can still strongly benefit from knowledge generated by others' programs.

With this in mind, we are developing a new model for data sharing for drug discovery for protozoan NTDs that will involve a loose consortium of NTD drug discovery labs who agree to confidentially share all of their data, models, and strategies as they are generated, within a group of other NTD-focused scientists [9]. As a “hybrid” arrangement of open and closed science, data (including compound structures and biological assessments) will be deposited in a password-protected database system (i.e. a “closed” model). However, the consortium will be open to any and all who are willing to agree to two primary terms: (1) real-time sharing of chemical structures and biological data; and (2) confidentiality with respect to data deposited in the consortium database. This kind of arrangement will not be a “public disclosure” per se (which would allow investigators to file patents if desired), yet will provide a mechanism for sharing data (positive and negative) with other NTD drug discovery researchers. This will create a new opportunity for sharing hypotheses, launching focused collaborations, and driving towards common target-product profiles for protozoan NTDs. In addition, we will provide an easy mechanism for participants to release their data to the general public when they are ready to do so (easily enabled by the Collaborative Drug Discovery vault we are utilizing [10]), and to draw in new experimental data for NTD research using an approach such as the Open Drug Discovery Teams project [11].

Some advantageous outcomes from such a collaboration could be

- Identification of synergistic directions to pursue in medicinal chemistry optimization;
- Reduction of unnecessary duplication of effort by reprioritization or termination of efforts on a given chemical series that others have shown to be intractable (inactive, toxic, insoluble), or to be currently under investigation in other labs for the same pathogen;
- We have observed our data-sharing methods to be a fruitful approach for anti-trypanosomal programs, for example, in which initial activity against *Trypanosoma brucei* was used to justify new programs against *Leishmania* and *T. cruzi* [12]. Our chemistry lab collaborates with several parasitology labs in *T. brucei, T. cruzi, Leishmania*, and *Plasmodium*, and most of these projects were launched with ideas spawned by sharing data across pathogens;
- Identification of similarities of drug scaffolds being evaluated across multiple groups, which could lead to efficient, ad hoc division of labor or scaffold-hopping campaigns [13]–[15];
- Open discussion of data in consortium meetings to generate new ideas and directions, and to inform each other's’ research programs;
- A clearing house for sharing preferred assay protocols, compound optimization endpoints, and computational models, as well as ideas, observations, and hypotheses;
- Opportunities for the consortium to partner with development organizations or contract vendors and perhaps become a clearinghouse for experimental suites that are favorably priced for NTD researchers;
- A large source of curated information that can be utilized by computational scientists to extract additional value from the data (predictive modeling, toxicity modeling, etc.);
- A mechanism by which positive and negative data can be released to the general public at the appropriate time, outside the framework of a traditional academic publication.

There are several considerations to evaluate at this point in implementing such a data sharing system. First, we need to assess the importance of intellectual property within the NTD drug discovery space. Scholarship, discussion, and action are needed in this area. Second, some modicum of funding will be needed to catalyze, grow, and nurture this type of consortium, such as to defray the cost of the database system, or to incentivize participation by funding experimental resources that are made available for consortium members. This is important, since such infrastructural undertakings are seldom attractive for funders, who typically wish to fund the research itself rather than a framework for facilitating research. Third, establishment of data sharing norms in this “gray area” implicit in this “hybrid model” will require collegial, constructive, and frank discussion to arrive at a reasonable solution that balances the desire for security with an aspirational goal of openness.

This is not intended to supplant public database systems such as PubChem or ChEMBL, which, while they may be utilized for pre-publication sharing of data, are presently not used in this way by collaborating NTD research groups. Indeed, the data deposited within this consortium will also be deposited into these public systems following publication or data release by the owning investigator.

In sum, it seems clear that a secure and collaborative data sharing model is needed, especially in the resource-limited research environment that is NTD drug discovery. While a fully open and coordinated drug discovery model for NTDs (perhaps modeled after OSDD-Malaria) should be an aspirational goal, there is a need for middle ground that will enable drug discovery scientists in the academic environment to more broadly and securely share data in real time. I posit that an opportunity therefore exists to enhance collaboration through secure data sharing prior to eventual open availability at the appropriate time.
